# Intrinsic neuronal resilience as a tool for therapeutic discovery

**DOI:** 10.1093/brain/awaf010

**Published:** 2025-01-15

**Authors:** Stefania Corti, Eva Hedlund

**Affiliations:** Dino Ferrari Centre, Neuroscience Section, Department of Pathophysiology and Transplantation (DEPT), University of Milan, 20122 Milan, Italy; Foundation IRCCS Ca’ Granda Ospedale Maggiore Policlinico, Neurology Unit, 20122 Milan, Italy; Department of Biochemistry and Biophysics, Stockholm University, 106 91 Stockholm, Sweden; Department of Cell and Molecular Biology, Karolinska Institutet, 171 77 Stockholm, Sweden

## Abstract

Corti and Hedlund argue that understanding the molecular underpinnings of neuronal resilience and vulnerability to neurodegenerative diseases such as ALS is key to identifying new therapeutic targets.

Neurodegenerative diseases are characterized by the selective loss of particular neuronal populations with resulting clinical features. In amyotrophic lateral sclerosis (ALS) the main symptoms are caused by the progressive loss of alpha motor neurons that innervate voluntary skeletal muscles throughout the body. The specialized synapses between motor neurons and muscle, the neuromuscular junctions (NMJs) are destroyed first, followed by distal to proximal axon degeneration and subsequent muscle atrophy and loss of motor neuron somas. Disease causative mutations in broadly or even ubiquitously expressed genes including *SOD1*, *FUS, C9ORF72* and *TARDBP* cause familial forms of ALS, where the proteins form aggregates and often mislocalize in the cells, affecting many cell types in the process. Nonetheless, it is motor neurons that handle the disease burden worst and die first. Why motor neurons degenerate is still a topic of intense investigation and seems related to their enormous energy demand, large size, extensive branching and connectivity, as well as vulnerability to endoplasmatic reticulium (ER) stress (Fig. 1).

**Figure 1:**
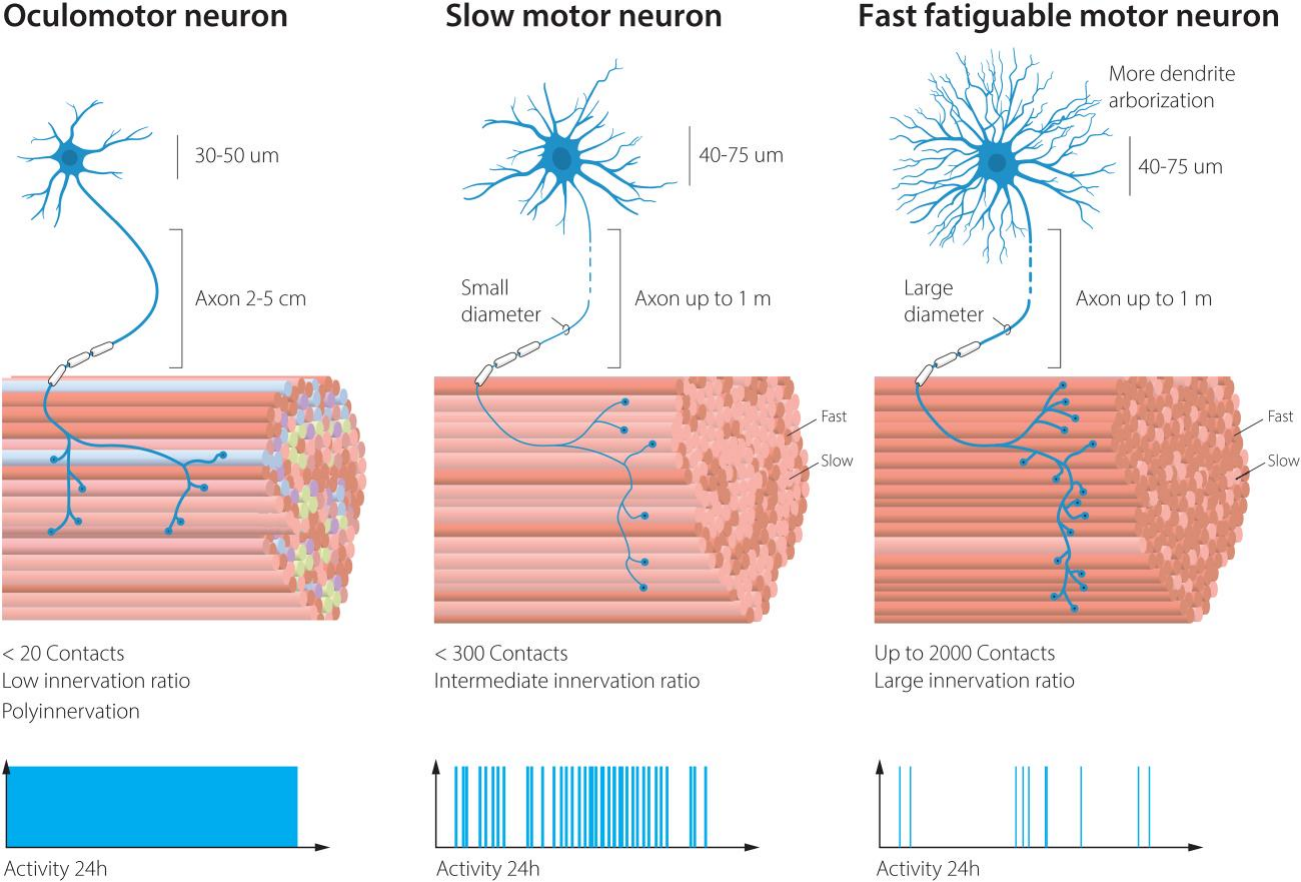
**Vulnerability to degeneration in ALS among motor neuron subpopulations is correlated to total volume of the cell, connectivity pattern and activity.** ALS = amyotrophic lateral sclerosis.

The selectivity and pattern of degeneration among motor neuron subpopulations have long intrigued researchers. The most vulnerable motor neurons in ALS are the fast fatigable, which are used during exertive movements such as jumping and sprinting, and which most people use only a few minutes per day. Fast fatigable motor neurons have enormous branching patterns into the muscle groups they control (Fig. 1). These neurons appear highly vulnerable to ER stress^1^ (Fig. 2A). Slow motor neurons are more resilient to ALS and even sprout to compensate for the loss of their fast fatigable motor neuron neighbours, occupying vacated endplates on muscle, rebuilding NMJs temporarily and causing muscle fibres to switch from a fast fatigable to a slow type in the process.^2^ Slow motor neurons are used ∼30% of our awake time and aid in keeping our posture and slower movements, such as walking and marathon running. Slow motor neurons have thinner axons than fast fatigable motor neurons and fewer connection points with the slow muscles they innervate and thus far smaller volumes than fast fatigable motor neurons, even if the targets they innervate are at a similar distance from the somas (Fig. 1). Among the most resilient of all somatic motor neurons are the oculomotor (OMNs) motor neurons (cranial nerves 3), which persist until end-stage of disease. These neurons regulate eye movements and are used constantly, and through their resilience to ALS, these are used to enable paralysed patients to communicate through ocular tracking devices. Thus, there is a direct correlation between neuronal activity and resilience. As OMNs have significantly shorter axons than fast fatigable and slow motor neurons and each neuron innervates only a few muscle fibres, there is also a correlation between the volume of the cell and resilience, as well as the number of targets the motor neuron innervates. However, these parameters do not provide a molecular explanation for the differences in intrinsic resilience mechanisms.

**Figure 2:**
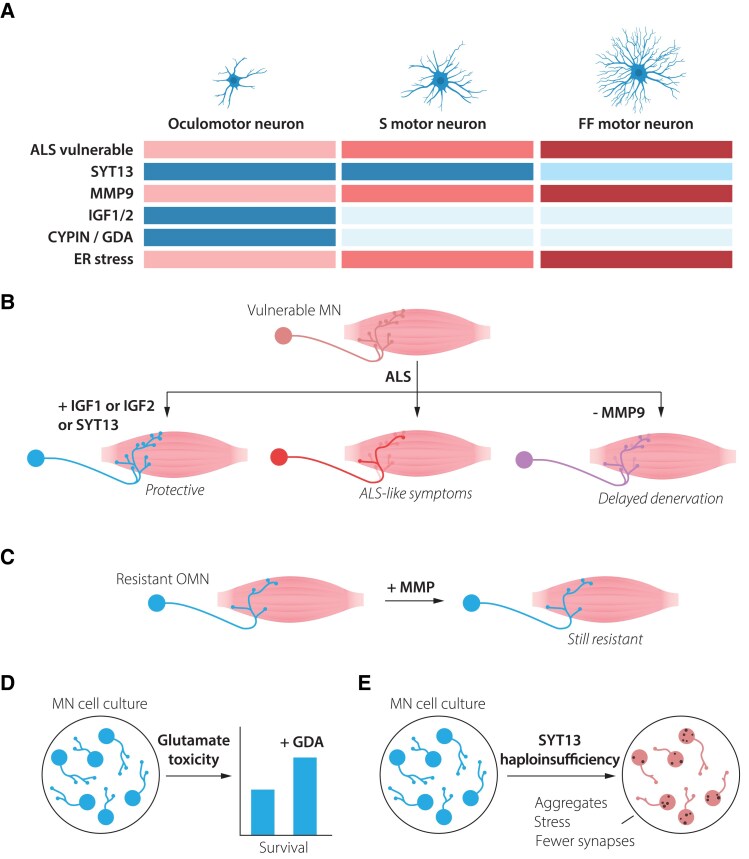
**Resistant motor neurons are defined by both the presence of resilience factors and the lack of vulnerability-inducing proteins**. (**A**) Oculomotor neurons show high resilience to amyotrophic lateral sclerosis (ALS), while slow motor neurons are more vulnerable and fast-fatiguable motor neurons show the highest vulnerability. Oculomotor neurons express high levels of several factors that may mediate their resilience, including SYT13, IGF-1, IGF-2 and CYPIN (GDA). They also lack MMP-9, which is expressed at high level in fast fatigable motor neurons and renders them vulnerable to ALS. Oculomotor neurons show low level of ER stress during ALS, while fast fatigable motor neurons demonstrate early and high ER stress levels. (**B**) Introduction of oculomotor-restricted genes into vulnerable spinal motor neurons can make these more resilient to degeneration in ALS. (**C**) ALS-resistent motor neurons, including oculomotor neurons and slow motor neurons maintain their resilience, even when the vulnerability factor MMP-9 is introduced to them. (**D**) Spinal motor neurons exposed to ALS-like glutamate excitotoxicity can partly be rescued by administration of GDA (CYPIN), which is normally expressed at high levels in resilient oculomotor neurons. (**E**) Motor neurons haploinsufficient for SYT13 show ALS-like phenotypes with inclusion of aggregates, increased stress and decrease in the number of synapses. ER = endoplasmatic reticulium; GDA = guanine deaminase; MN = motor neuron; OMN = oculomotor neuron.

ALS is in many ways a systems-biology disease, with involvement of a multitude of cell types. Nonetheless, it is evident that disease can be initiated from neurons alone and that differential vulnerability can be a cell intrinsic process. For example, when TAR DNA-binding protein-43 (TDP-43) with a defective nuclear localization signal was conditionally overexpressed in neurons alone, this caused selective motor neuron degeneration, with OMNs remaining intact and fast fatigable motor neurons showing higher vulnerability than slow. This intrinsic difference seemed related both to the ability of OMNs to better retain endogenous TDP-43 in the nucleus, compared to the vulnerable motor neuron populations, as well as an ability to handle mislocalized cytoplasmic TDP-43.^3^

The differential vulnerability among motor neurons holds crucial clues for understanding disease mechanisms and developing targeted therapies.^3-7^ Thus, there has been extensive research conducted to molecularly define the intrinsic properties of motor neuron subpopulations and thus deduce what factors make these more or less resilient to degeneration. As detailed below, ALS-resilient motor neurons appear to rely on the coordinate action of multiple protective mechanisms that work in concert. For instance, OMNs naturally produce high levels of the neurotrophic factors IGF-1 and IGF-2 and show elevated activation of PI3K-Akt signalling^4^ (Fig. 2A), which in many cases is necessary for trophic factor-induced cell survival. Overexpression of either IGF-1 or IGF-2 in susceptible motor neurons, using adeno-associated virus, protect these from degeneration in ALS mouse models and prolonged lifespan, while inducing peripheral nerve sprouting and formation of new NMJs.^4^ IGF-2 was also shown to protect human stem cell-derived motor neurons across ALS-like conditions (Fig. 2C). IGF-1 has been used in a clinical trial in ALS, but only using subcutaneous delivery (one or two doses), with contradictory results. We believe the lack of success could be explained by the delivery route, in which the molecule is unlikely to ever reach its therapeutic targets or their connected muscle fibres.

Stem cell-derived OMNs grown in a dish also appear intrinsically more resilient to ALS-like toxicological stress in the form of kainic acid, than spinal motor neurons,^5^ which may in part be due to their higher GABA-mediated chloride current and reduced inward calcium current, mediated through AMPA receptors. This resilience may also, in part, be related to protective IGF-signalling as *in vitro*-derived OMNs, similarly to their *in vivo* counterpart, appear to have naturally high activation of PI3K-AKT signalling compared to spinal motor neurons, as shown through pAKT and β-catenin levels.^5^ AKT signalling may also promote survival by inhibiting p53, which induces cell death genes and is highly activated across ALS causations. AKT is also thought to act by increasing ATP levels and glucose levels, allowing energy balance to tip towards survival. As ALS is thought to go through a hypermetabolic state, this may be another way for neurons to protect themselves.

CYPIN (or guanine deaminase, GDA) a protein important for dendritic branching and synaptic function, also shows a higher baseline expression in OMNs than spinal motor neurons and could protect spinal motor neurons from excitotoxicity when added to these^6^ (Fig. 2D). This indicates that OMNs have several lines of defence to stressors that other motor neurons succumb to.

Fast fatigable motor neurons—on the other hand—contain relatively high levels of, for example, the metalloproteinase MMP-9, which renders them vulnerable (Fig. 2A). Neuronal MMP-9 participates in synaptic plasticity likely through cleaving ECM components and cell adhesion molecules, including ICAM-5 and Neuroligin-1, controlling the function of excitatory synapses and shape of dendritic spines. MMP-9 also promotes inflammation. Interestingly, forced expression of MMP-9 was insufficient to trigger degeneration of OMNs or slow motor neurons in SOD1G93A mice, while its further overexpression in fast fatigable motor neurons induced their demise even faster^7^ (Fig. 2C). Thus, it appears that OMNs both lack certain factors that induce susceptibility as well as contain multiple factors and pathways that promote their resilience, even when extensively challenged.

Among several molecular characteristics that distinguish resistant OMNs from vulnerable motor neurons are their relatively high expression of the atypical synaptotagmin (SYT), *SYT13*, which appears broadly neuroprotective^8^ (Fig. 2A). *SYT13* gene therapy targeted to vulnerable motor neurons prolonged the lifespan of SOD1G93A ALS mice as well as spinal muscular atrophy (SMA) mice, by protecting the neurons and rescuing their connectivity with muscle (Fig. 2B). Human motor neurons were also protected *in vitro* across motor neuron disease toxicities and mutations, and while it is not clear how SYT13 works mechanistically, it was shown that the treatment reduced ER stress and apoptosis.^8^ RNA sequencing analysis of human post-mortem tissues of end-stage ALS patients, demonstrated that *SYT13* levels were as high in remaining spinal motor neurons as in OMNs. As motor neurons were sequenced in pools, it could at that time not be resolved if remaining relatively resilient spinal motor neurons that expressed high levels of SYT13 were of a slow type. Recent studies with single cell resolution have shed further light on this matter, with fluorescent *in situ* hybridization (FISH) experiments demonstrating that *Syt13* mRNA levels were higher in smaller (presumed slow) than in larger (presumed fast fatigable) motor neurons in healthy mice.^9^ Single nuclei RNA sequencing from mouse spinal cord further supports this conclusion^10^ with the level of *Syt13* correlating with the slow motor neuron marker *Sv2a*, but not with the fast fatigable motor neuron marker *Kcnq5* (http://alphamns.spinalcordatlas.org/) (Supplementary Fig. 1). Thus, all relatively resilient motor neurons appear to contain higher baseline levels of *SYT13* than their vulnerable counterparts, tying together a resilience factor across motor neuron populations in brainstem and spinal cord. This finding^10^ exemplifies how single nuclei approaches can provide unprecedented resolution and aid in understanding the molecular signatures of different motor neuron populations.

Furthermore, *SYT13* haploinsufficiency in human induced pluripotent stem cell (iPSC)-derived motor neurons triggered neurodegenerative phenotypes reminiscent of ALS, including protein aggregation, increased cellular stress and synaptic loss^9^ (Fig. 2E). These findings tie together the documented lower ER stress tolerance of fast fatigable motor neurons in ALS^1^ with SYT13 expression levels. It also indicates that a certain threshold of SYT13 expression is not only sufficient to protect motor neurons,^8^ but also required to maintain them.^9^ While SYT13 represents an important protective factor, it likely functions within a broader network of cellular mechanisms that collectively determine motor neuron fate in disease. It remains to be investigated if haploinsufficiency of SYT13 in OMNs would render these vulnerable too. As OMNs appear less sensitive to protein misfolding stress than spinal motor neurons, this indicates that OMNs have a toolbox to keep stress in check, which may in part be mediated by SYT13. A striking aspect is SYT13’s protective effects across different genetic causes of motor neuron disease. This broad efficacy suggests that SYT13-based therapies could potentially benefit a wide range of patients, regardless of the specific genetic mutations driving their disease.

In conclusion, recent research highlights the importance of investigating resistant motor neuron populations, such as OMNs, and slow motor neurons in ALS, and comparing them with vulnerable counterparts.^4-8^ This approach can uncover factors that provide insight into disease mechanisms and identify potential therapeutic targets. Looking ahead, single nucleus RNA sequencing has the potential to provide even further insights into the molecular complexity underlying selective vulnerability. We anticipate that intrinsic properties of fast fatigable and slow spinal motor neurons will be further dissected, as well as their response to ALS. We also expect that spatial sequencing methods, which are now at the capacity of organelle resolution, will further resolve subcellular distribution and quantity of particular signalling molecules, better revealing how these confer protection or cause detriment.

Thus, the technological breakthroughs are likely to deepen our understanding of the complex interplay between vulnerability and resilience factors, potentially revealing new therapeutic targets for ALS.

## Supplementary Material

awaf010_Supplementary_Data

## Notes

Dr Stefania Paola Corti is a Professor of Neurology at the University of Milan and Director of the Neuromuscular and Rare Diseases Unit at Fondazione IRCCS Ca' Granda Ospedale Maggiore Policlinico di Milano, Italy. Her research focuses on translational medicine and the development of novel cellular and molecular therapies for neurodegenerative diseases, particularly motor neuron disorders.

Dr Eva Hedlund is a Professor of Neurochemistry at Stockholm University in Sweden. Her research is focused on elucidating mechanisms of neuronal vulnerability and resistance with the goal of identifying new molecular targets for the treatment of neurodegenerative diseases, with a particular emphasis on amyotrophic lateral sclerosis (ALS).
