# Supplementary material for: Intrinsic neuronal resilience as a tool for therapeutic discovery
Source: Brain. 2025 Jan 15;148(4):1058–61. doi: 10.1093/brain/awaf010 (PMC11967791; doi:10.1093/brain/awaf010)
Supplement: awaf010_Supplementary_Data [file awaf010_supplementary_data.pdf]

Supplemental Figure 1. *Syt13* expression aligns more closely with the slow motor neuron marker *Sv2a* rather than with the fast fatiguable motor neuron marker *Kcnq5 (data from* http://skeletalmns.spinalcordatlas.org/ *and http://alphamns.spinalcordatlas.org/)*.

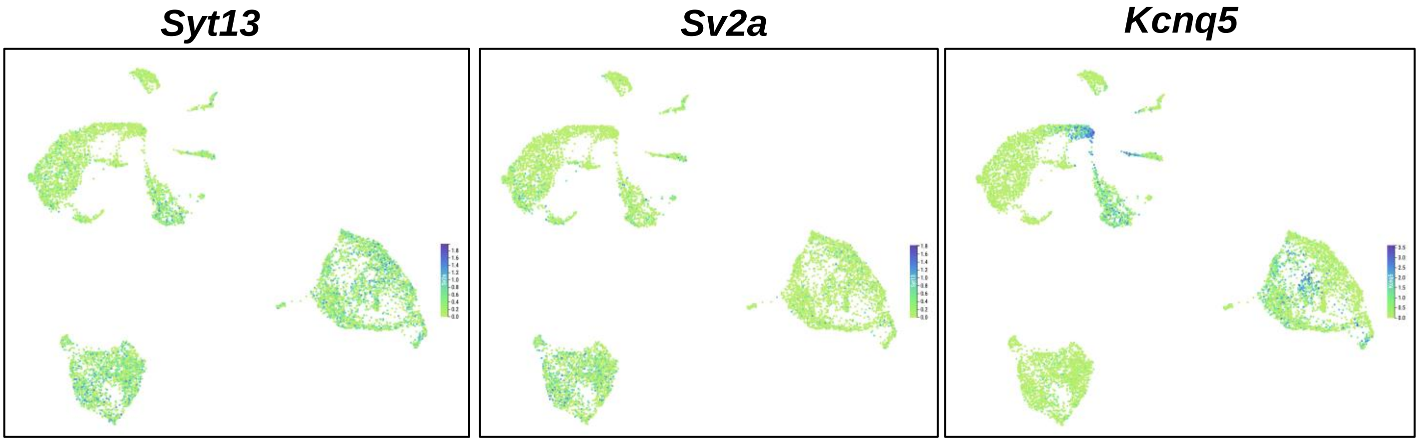

Analysis among skeletal motorneuron: http://skeletalmns.spinalcordatlas.org/

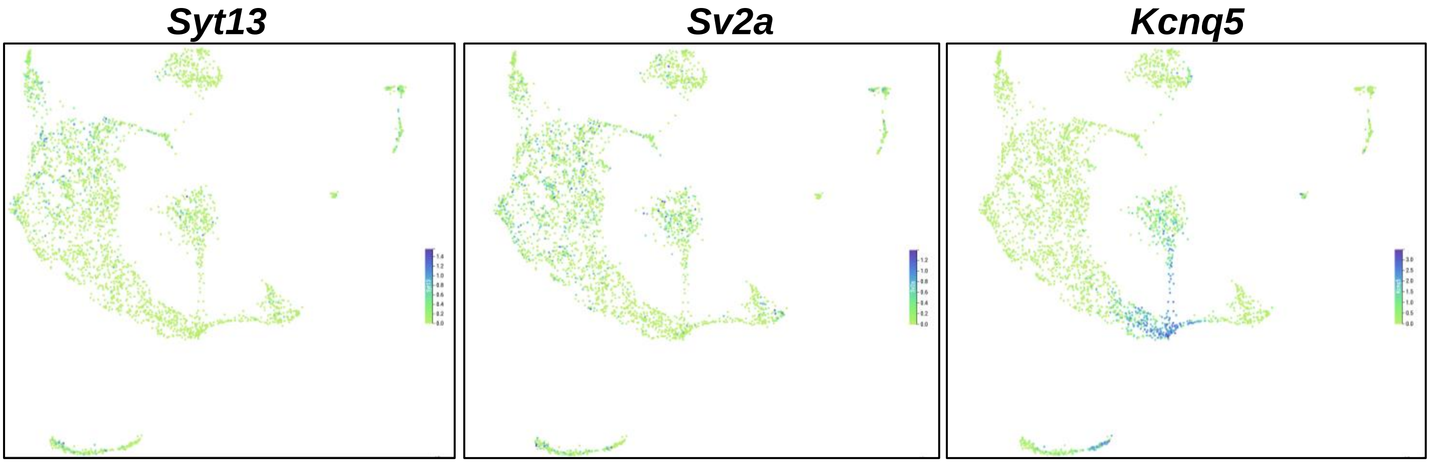

Analysis among alpha motor neurons: http://alphamns.spinalcordatlas.org/
